# Neurological Characteristics of Pediatric Glycogen Storage Disease

**DOI:** 10.3389/fendo.2021.685272

**Published:** 2021-05-21

**Authors:** Julio Henrique Muzetti, Daniel Almeida do Valle, Mara L. S. Ferreira Santos, Bruno Augusto Telles, Mara L. Cordeiro

**Affiliations:** ^1^ Faculdades Pequeno Príncipe, Curitiba, Brazil; ^2^ Instituto de Pesquisa Pelé Pequeno Príncipe, Curitiba, Brazil; ^3^ Department of Child Neurology, Hospital Pequeno Príncipe, Curitiba, Brazil; ^4^ Department of Radiology, Hospital Pequeno Príncipe, Curitiba, Brazil; ^5^ Department of Psychiatry and Biological Behavioral Sciences, University of California Los Angeles, Los Angeles, CA, United States

**Keywords:** glycogen storage disease, phosphorylase kinase deficiency, glucose-6-phosphatase deficiency, central nervous system, hypoglycemia, brain injury, mutation

## Abstract

Glycogen storage diseases (GSD) encompass a group of rare inherited diseases due dysfunction of glycogen metabolism. Hypoglycemia is the most common primary manifestation of GSD, and disturbances in glucose metabolism can cause neurological damage. The aims of this study were to first investigate the metabolic, genetic, and neurological profiles of children with GSD, and to test the hypothesis whether GSD type I would have greater neurological impact than GSD type IX. A cross-sectional study was conducted with 12 children diagnosed with GSD [Types: Ia (n=5); 1, Ib (n=1); 4, IXa (n=5); and 1, IXb (n=1)]. Genetic testing was conducted for the following genes using multigene panel analysis. The biochemical data and magnetic resonance imaging of the brain presented by the patients were evaluated. The criteria of adequate metabolic control were adopted based on the European Study on Glycogen Storage Disease type I consensus. Pathogenic mutations were identified using multigene panel analyses. The mutations and clinical chronology were related to the disease course and neuroimaging findings. Adequate metabolic control was achieved in 67% of patients (GSD I, 43%; GSD IX, 100%). Fourteen different mutations were detected, and only two co-occurring mutations were observed across families (*G6PC* c.247C>T and c.1039C>T). Six previously unreported variants were identified (5 *PHKA2*; 1 *PHKB*). The proportion of GSD IX was higher in our cohort compared to other studies. Brain imaging abnormalities were more frequent among patients with GSD I, early-symptom onset, longer hospitalization, and inadequate metabolic control. The frequency of mutations was similar to that observed among the North American and European populations. None of the mutations observed in *PHKA2* have been described previously. Therefore, current study reports six GSD variants previously unknown, and neurological consequences of GSD I. The principal neurological impact of GSD appeared to be related to inadequate metabolic control, especially hypoglycemia.

## Introduction

Glucose is stored in the form of glycogen, primarily in the cytoplasm of liver and muscle cells, and to a lesser degree, in brain tissues (1, 2). Hepatic glycogen plays a critical role in maintaining glucose homeostasis (1, 3). Inherited abnormalities in enzymes and regulators involved in the glycogen synthesis and degradation pathways cause a rare group of metabolic conditions known as glycogen storage diseases (GSD) (1, 3).

Twelve types of GSD have been identified, which are classified according to their associated enzyme deficiencies (4, 5). The overall incidence of GSD is estimated to be 1:10,000 live births (5). Subtypes I and III, are the most common type, whereas subtype IX are considered to be rare and it prevalence remain to be estimated (1). The clinical manifestations of GSDs vary according to the defective enzyme and its relative expression in different tissues, especially the liver and skeletal muscle (4, 6). GSDs with liver involvement (hepatic GSDs) are a complex group of disorders, the main symptoms of which include hypoglycemia and hepatomegaly (1, 5).

GSD type I occurs due to a deficiency of the enzyme glucose-6-phosphatase α (G6Pase α), which impairs the ability to produce glucose *via* glycogenolysis and gluconeogenesis. This enzyme is anchored onto the ER and catalyzes the conversion of G6P to glucose and inorganic phosphate. Two main subtypes of GSD I are recognized: GSD type Ia (GSDIa; OMIM #232200) and GSD type Ib (GSDIb; MIM #232220). GSD Ia results from a mutation in the *G6PC* gene (MIM *613742), which encodes the catalytic subunit of G6Pase α. GSD Ib results from a biallelic pathogenic variant in the *SLC37A4* gene (MIM *602671), which codes for a G6P translocase. A defect in this translocase’s activity accounts for about 10% of the cases (7, 8).

GSD type IX results from a deficiency of hepatic phosphorylase kinase (PhK), the enzyme responsible for the activation of glycogen phosphorylase and a key controller in the mobilization of glucose from glycogen. The PhK enzyme is comprised of four copies of each of the four subunits (α, β, γ, and δ), encoded respectively by the genes *PHKA1*, *PHKA2*, *PHKB*, and *PHKG2*. GSD IXa (MIM #306000) is an X-linked recessive disorder caused by a pathogenic mutation in the *PHKA2* gene (MIM *300798); GSD IXb (MIM #261750) is caused by a compound heterozygous mutation in the *PHKB* gene (MIM *172490) (9, 10). The most common clinical manifestations of GSD type IX include hepatomegaly, elevated liver enzymes, and short stature (9, 11).

The dysregulation of glucose metabolism affects the entire central nervous system and can cause serious damage, because glucose is essential for normal neuronal function (4). Low brain glycogen reserves hinder local glucose availability as a conventional energy source, causing a continuous demand for glucose from the circulation and ultimately using approximately 25% of the body’s glucose (2, 12). The tricarboxylic acid cycle ceases to function during hypoglycemic episodes, leading to increased levels of glutamate and aspartate, which affects the sodium/water balance and can therefore cause cellular edema (12). Studies using animal and neurofunctional models have shown that moderate intermittent hypoglycemia has a direct effect on the function of the hippocampus, by reducing its volume and altering neuronal synapses (13, 14).

Disorders of glycogen metabolism are associated with liver and muscle disorders. Although the presence of glycogen in the brain has been recognized for decades, its functional roles in the brain have been discovered only recently (2, 7), including the profound molecular contributions of glycogen metabolism to the brain (7). Brain glycogen may act as an energy substrate during periods of increased energy demand, such as learning and memory processes (2). Therefore, we aimed to investigate the biochemical and genetic aspects of GSD in Brazilian pediatric patients and test the hypothesis that GSD I would have greater impact on the brain than other types of GSD such as the X-linked GSD type IX.

## Methods

### Study Design and Participants

This cross-sectional, observational, descriptive study enrolled children diagnosed with GSD who were being followed-up at the rare diseases outpatient clinic of the *Hospital Infantil Pequeno Príncipe*, Curitiba, PR, Brazil.

The study was conducted between January 2020 and January 2021. A convenience sampling strategy was used. All participants were assessed by the same researcher, who conducted targeted history taking and physical examination. The variables of interest included sex, current age, age at diagnosis, duration of hospitalization due to GSD (in days), and current laboratory serum levels. All patients had their diet with energy needs assessed individually and proportions of diet components introduced once the diagnosis was established.

The inclusion criteria were as follows: patients aged 2–14 years old, with a clinical diagnosis of GSD, requiring ≥12 months of specialized care, who presented with the clinical manifestations of GSD, including hypoglycemia, hyperlactatemia, hypertriglyceridemia, hyperuricemia, hepatomegaly, and/or a growth deficit (short stature for age) at the time of diagnosis or inclusion in the study. Patients with associated neurodegenerative diseases were excluded.

### Measures

Serum levels were recorded from each patient’s most recent medical record. The biochemical blood parameters evaluated included blood glucose, triglycerides, high-density lipoprotein, low-density lipoprotein, cholesterol, uric acid, and lactate. Since, in their totality, patients showed adequate adherence to the diet, based on direct recall or use of food diaries, the evaluation of dietary control was carried out through metabolic criteria. The following criteria of adequate metabolic control were adopted based on the European Study on Glycogen Storage Disease type I (ESGSD I) consensus: glucose >63 mg/dL, triglycerides <530 mg/dL, uric acid <7 mg/dL, lactate <2.5 mg/dL, and body mass index within 2 standard deviations from the population mean (6, 13).

Genetic testing was conducted for the following genes using multigene panel analysis: *AGL, FBP1, G6PC, GAA, GBE1, GYS2, PHKA2, PHKB, PYGL*, *SLC2A2*, and *SLC37A4*. Buccal swab samples were collected, and DNA was extracted for genetic analyses of the target genomic regions. Next-generation sequencing was performed using Illumina technology: alignment and variant identification was performed based on bioinformatics protocols using the GRCh38 human genome as a reference. The potential pathogenic variants and regions with inadequate sequencing depth were confirmed using automated Sanger sequencing, which was conducted with a genetic analyzer. The variants were described according to the nomenclature recommended by the Human Genomic Variation Society.

Novel variants were classified according to the guidelines of the American College of Medical Genetics and Genomics (15) on the basis of very low allele frequency, compound heterozygosity with a pathogenic variant, residue evolutionary conservation, and biochemical results. New variants were deposited in the Human Gene Variant Database (https://www.hgmd.cf.ac.uk/) and ClinVar database (https://www.ncbi.nlm.nih.gov/clinvar/). Mutations were grouped according to type (missense or non‐missense). Mutations resulting in a frameshift or splicing modifications were considered to be potentially pathogenic. The pathogenicity of novel missense mutations was predicted using in-silico analyses.

### Magnetic Resonance Imaging

Brain MRI datasets obtained within the 5 years prior to the study were analyzed retrospectively. If the patient had more than one neuroimaging scan, the images were compared to assess the appearance of new lesions. MRI was requested for patients who had not undergone neuroimaging during the past 5 years. Brain MRI was performed with a 1.5-T magnetic resonance unit (Signa Explorer, GE Medical Systems, Milwaukee, WI). T1-weighted [echo time (TE)/repetition time (TR), 11 ms/550 ms], T2-weighted (TE/TR, 93 ms/4000 ms), fluid-attenuated inversion recovery (TE/TR/inversion time, 110 ms/10000 ms/2250 ms), and diffusion-weighted (TE/TR, 105 ms/5200 ms) imaging was performed.

### Statistical Analyses

Data were stored and analyzed using Microsoft Excel 2016 and SPSS for Windows (v. 22.0, IBM, Armonk, NY). Descriptive analyses were performed by calculating the summary measures. Inferential analyses were performed using the Chi-squared, Fisher’s exact (non-parametric variables), and Student’s t tests (parametric variables), with a significance level of *p* < 0.05.

## Results

### Demographic and Clinical Findings

**Table 1** describes the participants’ clinical characteristics. This study enrolled 12 patients (age range, 2–17 years) of both sexes (9 boys and 3 girls) who were diagnosed with hepatic GSD, including 5 patients with GSD Ia, 1 with GSD Ib, 5 with GSD IXa, and 1 patient with GSD IXb. The chronology of each patient’s diagnosis and treatment by GSD type (I or IX) are presented in **Table 2**. The patients’ blood biochemistry test results classified by GSD type are presented in **Table 2**. Adequate metabolic control was achieved in 7/12 patients (58%) overall according to the ESGSD I criteria, including 2/6 patients (33%) with GSD I and 5/6 patients (83%) with GSD IX.

**Table 1:** Characteristics of glycogen storage disease (GSD) patients analyzed in this study.

Patient	Sex	GSD type	Age at symptom onset (months)	Age at diagnosis (months)	Adequate metabolic control*	Alleles	Normal Brain MRI
**1**	M	Ia	10	10	N	c.508C>T c.508C>T	N
**2**	M	Ixb	8		Y	c.352G>C c.570_576delinsAC	Y
**3**	F	Ia	28	40	N	c.1039C>T c.1039C>T	Not performed
**4**	M	IXa	0	0	Y	c.537+3_537+4insT	Y
**5**	M	IXa	19	45	Y	c.2735T>C c.2785G>C	Y
**6**	F	IXa	39	46	Y	c.277A>G	Y
**7**	M	IXa	35	48	N	c.1499G>A	Y
**8**	M	IXa	37	45	Y	c.537+3_537+4insT	Y
**9**	F	Ib	6	9	N	c.703_705delGTG c.1042_1043delCT	N
**10**	F	Ia	0	5	N	c.509G>A c.247C>T	N
**11**	M	Ia	17	17	Y	c.1039C>T c.3018+3C>G	Y
**12**	M	Ia	0	12	Y	c.247C>T c.247C>T	N

*Adequate metabolic control was defined based on the European Study on Glycogen Storage Disease type I (ESGSD I) consensus.

**Table 2:** Comparison of patients with glycogen storage disease types I and IX.

Patient characteristic	Mean (standard deviation)			*P*
	GSD I	GSD IX		
Age of symptom onset, months	10.2 (11.1)		23.0 (16.4)	0.150
Age at diagnosis, months	15.5 (12.5)		37.0 (20.7)	0.086
Total hospitalization time, months	22.3 (16.5)		5.7 (4.8)	0.039*
**Variable**				
Uric acid, mg/dL	7.5 (3.3)		3.9 (1.0)	0.068
Lactate, mg/dL	6.4 (3.6)		1.6 (0.3)	0.031*
Triglycerides, mg/dL	413.2 (323.6)		94.0 (38.9)	0.037*
High-density lipoprotein, mg/dL	36.2 (19.0)		49.4 (13.0)	0.236
Low-density lipoprotein, mg/dL	132.9 (30.2)		98.2 (14.3)	0.056
Blood glucose, mg/dL	74.0 (17.8)		71.8 (18.3)	0.839

*p < 0.05.

### Genetic Findings

**Table 3** describes the mutations identified in this study cohort as well as the biochemical consequences of these mutations. Fourteen different mutations were detected in four genes, including 5 in *G6PC* (17q21.31), 2 in *SLC37A4* (11.q23.3), 5 in *PHKA2* (Xp22.13), and 2 in *PHKB* (16q12.1). The majority of the mutations were of the missense/nonsense type and located in the exon regions. All mutations, except for two, were observed in a single family; two *G6P6* mutations were identified in two families each (c.247C>T and c.1039C>T). All five *PHKA2* variants and one of the two *PHKB* variants have not been reported previously.

**Table 3:** Summary of glycogen storage disease-related variants identified.

Gene	Type	Allele	Position	ACMG	Protein	No. mutations (family)
*G6PC*	Mis-/nonsense	c.247C>T	E2	Pathogenic	p.Arg83Cys	3(2)
		c.508C>T	E3	Pathogenic	p.Arg170*	2(1)
		c.509G>A	E3	Pathogenic	p.Arg170Gln	1(1)
		c.1039C>T	E5	Pathogenic	p.Gln347*	3(2)
	Splicing	c.3018+3C>G	I4	Pathogenic		1(1)
*SLC37A4*	Deletion	c.703_705delGTG	E5	Probably pathogenic	p.Val236del	1(1)
		c.1042_1043delCT	E5	Pathogenic	p.Leu348Valfs*53	1(1)
*PHKA2*	Mis-/nonsense	c.277A>G	E3	Uncertain	p.Met93Val	1(1)
		c.1499G>A	E15	Uncertain	p.Arg500Gln	1(1)
		c.2735T>C	E25	Uncertain	p.Met912Thr	1(1)
		c.2785G>C	E25	Uncertain	p.Ala929Pro	1(1)
	Splicing	c.537+3_537+4insT	I5	Probably pathogenic		2(1)
*PHKB*	Mis-/nonsense	c.352G>C	E5	Pathogenic	p.Ala118Pro	1(1)
	Deletion	c.570_576delinsAC	E6	Pathogenic	p.Gln191Hisfs*5	1(1)

*Mutations in red are not previously described.

### Magnetic Resonance Imaging Findings

**Figure 1:**
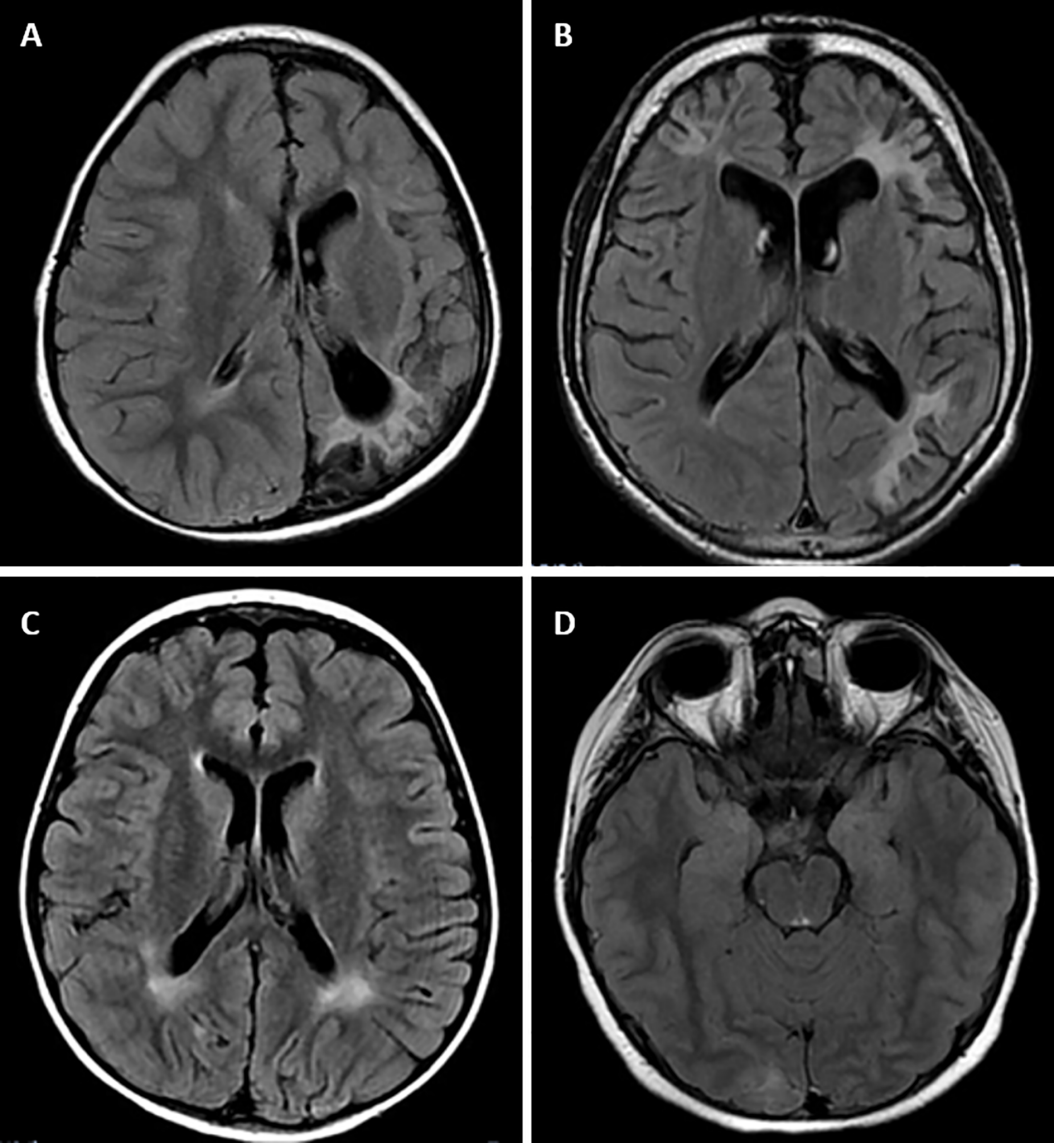
Magnetic resonance imaging alterations in patients with GSD Ia. **(A)** (Patient #1). Extensive areas of gliosis and encephalomalacia are observed, particularly in the cortical and subcortical areas of the left occipital and parietal lobes, as well as along the frontoparietal transition with high convexity and compensatory ectasia of corresponding portions of the ipsilateral lateral ventricle. Moreover, lesion foci are observed, which are probably related to hemosiderin deposits, extending to the posterior aspects of the nucleocapsular region with signs of chronic Wallerian degeneration of the corresponding cortico-spinal tract. There is substantial left-sided cerebral atrophy. **(B)** (Patient #12) Retracted lesions affecting the cortex and subcortical white matter of the bilateral frontal regions and the left parieto-temporal transition are evident. These changes are suggestive of encephalomalacia and vascular sequelae. **(C)** (Patient #9) Oval foci showing high-intensity on T2-weighted sequences in the central white matter and projecting close to the peritrigonal regions. These nonspecific changes could represent gliosis around the perivascular spaces (terminal myelination areas). **(D)** (Patient #10) Small retracted lesions affecting the posterior occipital poles with loss of volume, especially on the right side, suggestive of lesions occuring secondary to hypoglycemia.

MRI alterations were observed in 4 of the 6 patients with GSD Ia. None of the patients with the other types of GSD had MRI manifestations. Subcortical white matter hyperintensities were observed in the occipital lobes of 2 patients (**Figures 1A, B**). One patient presented with T2 hyperintense oval foci located in the central white matter that extended toward the peritrigonal regions (**Figure 1C**). One patient had retracted lesions affecting the cortex and subcortical white matter of the bilateral frontal regions and left parieto-temporal transition (**Figure 1D**). The lesion extent was highly variable for each individual. Neuroimaging abnormalities were more frequent among patients with early onset of symptoms (*p* = 0.010), longer hospitalization (*p* = 0.002), and inadequate metabolic control, and were significantly associated with uricemia (*p* = 0.043), hyperlactatemia (*p* = 0.001), hypertriglyceridemia (*p* < 0.001), and elevated low-density lipoprotein levels (*p* = 0.031) (**Table 4**).

**Table 4:** Magnetic resonance imaging (MRI) alterations relative to diagnosis, chronology variables, and metabolic variables.

Variable	Normal RI	Abnormal MRI	*p*
**Diagnosis, N**			
** GSD I**	2	4	0.061
** GSD IX**	5	0	
**Chronology, mean (standard deviation)**			
** Age of symptom onset, months**	22.98 (16.44)	5.24 (4.08)	0.010
** Age at diagnosis, months**	36.96 (20.71)	9.37 (0.85)	0.189
** Total hospitalization time, days**	6.86 (5.37)	26.25 (19.82)	0.002
**Adequate metabolic control** ^**a**^, **N**	7	1	0.024
**Metabolic index, mean (standard deviation)**			
** Uric acid, mg/dL**	4.08 (0.91)	8.73 (3.85)	0.043
** Lactate, mg/dL**	1.55 (0.34)	5.88 (3.85)	0.001
** Triglycerides, mg/dL**	89.71 (37.28)	439.00 (336.00)	<0.001
** High-density lipoprotein, mg/dL**	49.4 (13.01)	37.75 (21.55)	0.499
** Low-density lipoprotein, mg/dL**	98.20 (14.25)	119.00 (33.94)	0.031
** Blood glucose, mg/dL**	71,86 (16,69)	79.00 (19.58)	0.525

a According to European Study on Glycogen Storage Disease type I criteria.

## Discussion

The present cross-sectional, observational, descriptive study investigated the metabolic, genetic, and brain MRI profiles of children with GSD. Our GSD cohort included thrice as many boys as girls. This difference may be attributed to the high incidence of GDS IXa among the participants, since GSD IXa is attributed to mutations of *PHKA2*, which is located on the X chromosome (1). GSD Ia was also highly prevalent in our sample, which is consistent with the literature (1, 8). However, although GSD III is generally considered to be the second most frequent type, no patient in the present study was diagnosed with GSD III. Conversely, although GSD IXa is considered to be a rare form of GSD [prevalence < 1:100,000 and approximately 50 cases described in the literature (1), one-third of the patients in our sample (5/12) were diagnosed with GSD IXa. It is possible that GSD IXa is underdiagnosed in general, perhaps due to its oligosymptomatic and variable presentation, infrequent hospitalization, and fewer alterations in the laboratory profile (10).

Early diagnosis and timely treatment have been shown to have a positive impact on patients’ quality of life and prognosis, and are associated with a reduced risk of complications (16). The patients with GSD I in our cohort tended to be younger at symptom onset by one year or more compared to their counterparts with GSD IX, and several patients experienced symptoms before 1 year of age, similar to previous studies (17).

The frequency of *G6PC* mutations observed in this study population was similar to that reported for Caucasians in the USA (18), northwestern Europe (17, 19), and Rio Grande do Sul, Brazil (20, 21), with the c.247C>T and c.1039C>T polymorphisms being the most common mutations. The c.508C>T mutation in *G6PC* was previously identified in Japanese (22) and Dutch (17) populations, in which it accounted for about 6% of the mutations identified, similar to its proportion in our population. The c.509G>A mutation in *G6PC* was previously identified in a Dutch population (17), but with a low frequency. Although the Brazilian population has considerable ancestral heterogeneity, which includes indigenous Amerindians and immigrants from different regions of Europe, Africa, and Asia, the European ethnicity is the most (substantially) prevalent ancestry in Brazil, especially in the southern states, including Paraná, where this study was conducted.

Four (belonging to 3 families) of the 5 patients with GSD IX in our sample had *PHKA2* mutations, including one female patient, despite the location of *PHKA2* on the X chromosome and the X-linked inheritance of GSD IXa. Some women may present with symptoms of GSD IXa, depending on their X chromosome inactivation pattern (10, 23). Missense mutations (c.537+3_537+4insT, c.1499G>A, c.2735T>C, c.2324A>G, and c.2785G>C) were found in 4 patients in our cohort. None of these missense mutations have been described previously in the literature, indicating the paucity of knowledge on the etiology of GSD IXa and possibility of underdiagnosis (10).

Several factors are related to neuronal death induced by hypoglycemia, and not just related to energy failure. There is an increase in glutamate induced by hypoglycemia, with a reduction in astrocytic glutamate reuptake and increased activation of aspartate receptors (24). In response to excitotoxicity, reactive oxygen species levels increase due to production of superoxide after oxidation of NADPH during glucose reperfusion, provoking neuronal cell death (24, 25). Zinc is a neuromodulator stored in pre-synaptic vesicles, and is released into the extracellular space in the presence of pathological events, such as hypoglycemia and hypoxia. Zinc alters several receptors, including NMDA, GABA-A and ATP, and is associated with neural death (24). Another mechanism causing neuronal death occurs through activation of Poly-ADP-Ribose Polymerase (PARP). While normal PARP activity facilitates DNA repair and prevent the exchange of chromatids, extensive damage from the sustained action of glutamate causes extensive activation of PARP and increases mitochondrial permeability and damage, culminating in cell death (24).

Despite the difficulty associated with identifying the exact topography of lesions caused by hypoglycemia, lesions in the frontal lobes, temporal lobes, and basal ganglia have often been described in patients with diabetes type I (26). Possible contributors to the location of damage include reduction in regional use of cerebral glucose or deficit of local expression of the glucose membrane-transporting proteins (24). Although Melis et al. (27) found that all of their patients with GSD exhibiting MRI abnormalities had occipital horn dilatation and/or hyperintensity of the subcortical white matter in the occipital lobes, only 50% (2/4) of the patients with MRI abnormalities in the present study presented with these findings. During the neonatal period, there is a high degree of synaptogenesis and axonal migration to the occipital lobe associated with high levels of aspartate stimulation of newly developed receptors for excitatory amino acids. Hypoglycemia can damage this process, causing selective death in the postsynaptic neurons (28). The other 2 patients had peritrigonal changes and lesions in the cortical and subcortical regions of the fronto-temporal cortex, similar to the findings of Aydemir et al. (4). The occasional damage to frontal areas in some neonates with severe hypoglycemia involves infarctions of the distal field, typical in ischemic conditions (29).These neuroimaging findings are consistent with the hypothesis that the principal neurological effect of glycogenosis is the result of inadequate metabolic control, especially with respect to hypoglycemia (4, 27). Interestingly, and not previously investigated, our study allowed the comparison of neuronal damage from GSD type I with that of GSD IX. Although our sample is small, we observed that the patients with GSD type Ia presented more cerebral damage than those with GSD IX; this could have been related to earlier onset of symptoms, longer hospitalization, inadequate metabolic control, and elevated lactate levels in the GSD I patients. Earlier investigations of brain morphology and function of patients with GSD I found that patients performed worse in neuropsychological tests than normal controls and had abnormal EEG patterns (27); similarly, the brain MRI in 57% of patients with GSD type I was altered. Certainly, additional investigations are needed to validate our findings, as well as to compare the impacts and evaluate the underlying mechanisms of each GSD type on the brain.

The characterizations of the natural history of rare diseases and their impact on patients and their families is always hampered by small sample sizes, which is among the noteworthy limitations of this study. Accordingly, the present characterizations were limited by our small sample population of 12 patients, which included only 7 patients with GSD I and 5 patients with GSD IX. Nevertheless, we were able to identify GSD-associated mutations even in this small cohort that have not been described in the literature previously, and one-third of the cases in our sample were diagnosed with GSD IXa, which is generally considered to be very rare subtype of GSD (30), suggesting the probability of its underdiagnosis.

In conclusion, GSD Ia was the most prevalent form of GSD, affecting half of the patients in the present cross-sectional, observational, descriptive study. Notwithstanding, we observed a higher than typically reported prevalence of GSD IX, probably because GSD IXa may be oligosymptomatic with infrequent hospitalizations and few laboratory alterations, which would result in underdiagnosis. The frequency of *G6PC* mutations was similar to that reported previously for North American and European Caucasian populations. Notably, we detected two *PHKB2* mutations that have not been described previously, suggesting the probability of inadequate identification and underdiagnosis of GSD IXa in Brazil. The observations of MRI alterations in regions of high metabolism, such as the frontal lobes, temporal lobes, and basal ganglia, are consistent with the hypoglycemia-induced origin of the lesions. Thus, the main neurological impact of glycogenosis is apparently related to inadequate metabolic control, especially that of hypoglycemia.

## Data Availability Statement

The data presented in the study are deposited in the BioProject repository, accession number PRJNA725850 and the new mutations detected were uploaded to the ClinVar database.

## Ethics Statement

All procedures performed involving human participants were conducted in accordance with the ethical standards of the institutional and/or national research committee and with the 1964 Helsinki Declaration and its later amendments or comparable ethical standards. This study was approved by this hospital’s Human Research Ethics Committee of Hospital Pequeno Principe (n. CAAE: 31888620.6.0000.0097). Written informed consent to participate in this study was provided by the participants’ legal guardian/next of kin. Written informed consent was obtained from the minor(s)’ legal guardian/next of kin for the publication of any potentially identifiable images or data included in this article. Informed consent was also obtained for the publication of the images in **Figures 1A–D**.

## Author Contributions

JM conceptualized the research, gathered and analyzed the data, and wrote the initial manuscript. DV helped with the data analyses and draft of the initial manuscript. BT reviewed the neuroimaging scans. MC coordinated the study, and revised and conducted critical reviews of the manuscript for key intellectual content. MS helped with the data analyses. All authors contributed to the article and approved the submitted version.

## Funding

This study was funded in part by the Coordenação de Aperfeiçoamento de Pessoal de Nivel Superior-Brazil (CAPES) conferred to JM and DV (Finance Code 001).

## Conflict of Interest

The authors declare that the research was conducted in the absence of any commercial or financial relationships that could be construed as a potential conflict of interest.
